# Practical neutron detection modalities for industrial use available at a time-of-flight small-angle scattering instrument

**DOI:** 10.1107/S1600576725004479

**Published:** 2025-07-02

**Authors:** Satoshi Koizumi, Kazuki Mita

**Affiliations:** a Faculty of Engineering Ibaraki University Ibaraki Japan; b CROSS Tokai Japan; Australian Centre for Neutron Scattering, ANSTO, Australia

**Keywords:** industrial use small-angle neutron scattering, SANS, neutron radiography, reflectivity, prompt γ-rays, multi-measurement

## Abstract

We report the latest advances of the iMATERIA instrument, namely, time-of-flight small-angle neutron scattering (SANS) specifically for measurements in the manufacturing and energy industries. Observations are available that are multi-scale (from a minimum *q*_min_ = 0.007 Å^−1^ to a maximum *q* = 30 Å^−1^), multi-time domain (>0.5 s) and multi-contrast by dynamical nuclear polarization. Multi-angle (or stereo) observation of film specimens can bridge between conventional SANS and reflectivity measurements. For multi-analysis, we have developed real-time simultaneous SANS and neutron radiography (NR), targeting polymer electrolyte fuel cells. In the future, we plan to establish simultaneous triple-analysis combining SANS, NR and prompt γ-ray analysis. By scanning with a beam of 1 mm^2^ in size, such analysis enables the mapping of structural parameters determined by SANS and elemental composition determined by prompt γ-ray analysis on the image obtained by NR.

## Abstract 2

Various neutron detection approaches for industrial use at the time-of-flight small-angle neutron scattering instrument iMATERIA (J-PARC) are described here.

## 1. Introduction

The history of small-angle scattering stretches back to Peter Debye (1933 ▸). Now, materials research in industry is widely supported by equipment ranging from laboratory-sized X-ray emission instruments to large-scale facilities using neutrons (Jeffries *et al.*, 2021 ▸). This is important because hierarchical structures exist from the atomic scale to the mesoscale, which is directly linked to the function of products. At an international conference on small-angle scattering, SAS2022 ▸, held in Brazil in 2022 ▸, Dr Andrew J. Allen (NIST) held a keynote lecture from the perspective of industrial use (Allen, 2023 ▸). He gave an introduction to recent progress in small-angle scattering instruments, comprehensively covering both X-ray and neutron beams. It was emphasized that, at pulsed neutron sources, small-angle neutron scattering (SANS) can be increasingly integrated with total scattering studies of local ordering in complex materials, or with neutron diffraction for engineering materials and alloys.

We have also been working on the observation of soft materials such as polymers while developing ultra-small-angle scattering instruments for both reactor and accelerator neutron sources (Agamalian & Koizumi, 2011 ▸; Koizumi & Noda, 2019 ▸; Koizumi *et al.*, 2007 ▸; Koizumi *et al.*, 2020 ▸). In this paper, we describe a number of industrial and environmental use cases, starting from a small-angle scattering instrument base, *i.e.* the Ibaraki Prefecture Material Structure Analysis Instrument (iMATERIA) (Koizumi *et al.*, 2020 ▸), dedicated to industrial use installed at a high-energy (1 MW) proton accelerator facility (J-PARC). Pulsed neutrons and the time-of-flight (TOF) data acquisition method have a rich vein of developments, including neutron diffraction, reflectivity, imaging (radiography), prompt γ-ray analysis, and image mapping of hierarchical structural and element information. J-PARC attained a thermal output of 850 kW in 2022 ▸, and the ultimate target of 1 MW is becoming a reality. Against this background, this paper describes the current status of the various industrial uses that are underway, giving examples from the iMATERIA instrument. The aim is to increase the efficiency of measurements through a diverse range of trials and to provide multi-faceted information for the diverse problems that arise in industry. The keyword for this paper is ‘multi-measurement’ for industry.

## 2. A variety of industrial uses on one beam of the iMATERIAL instrument at J-PARC

What kind of experiments can be performed by using the high energy of J-PARC, especially when employing powder diffraction at the same beamline? The following presents examples of the achievements of iMATERIA (BL20).

### 2.1. Multi-scale observation

**Figure 1:**
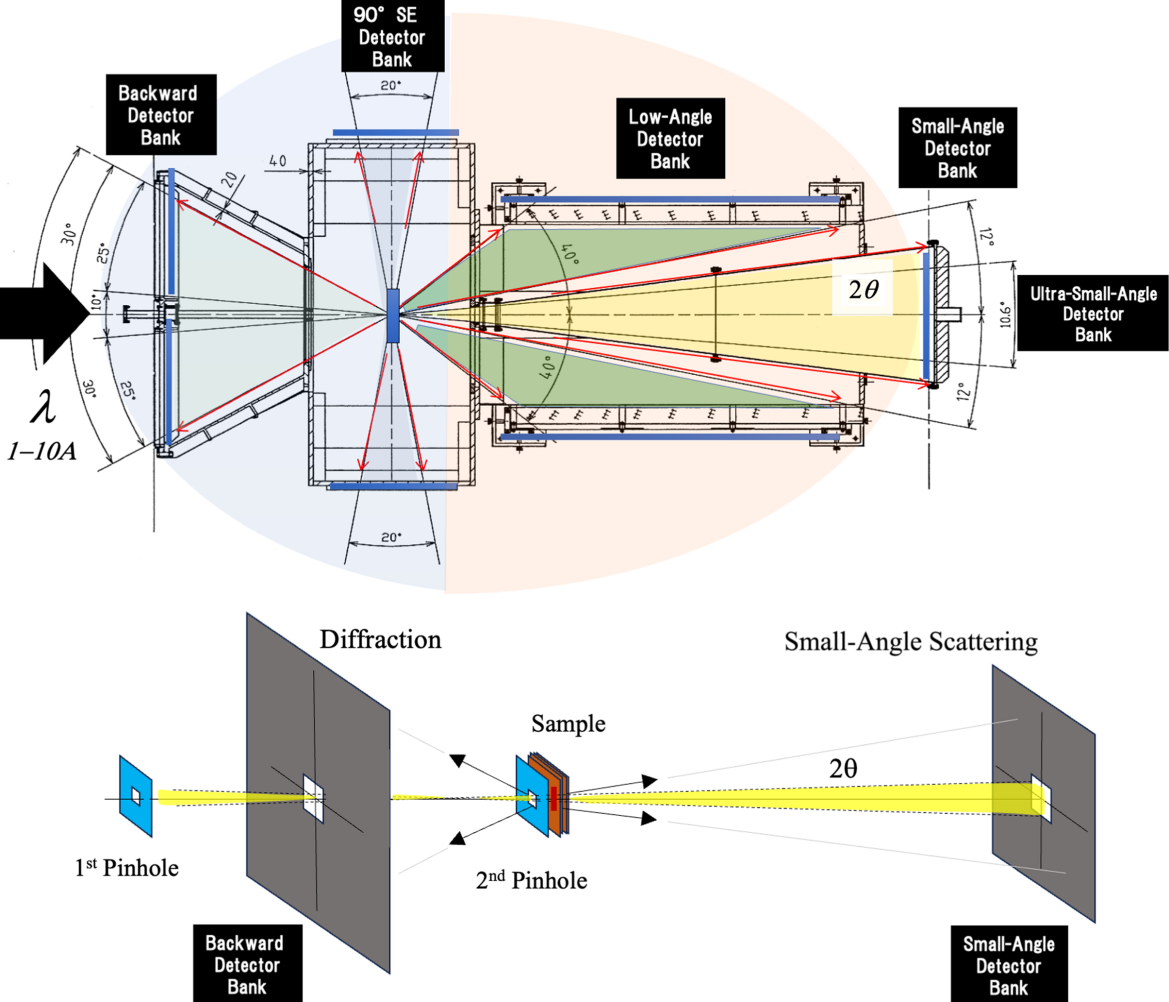
(Top) Schematic of the iMATERIA instrument, showing four detector blocks for small-angle, low-angle, 90° and backward detectors. (Bottom) Simultaneous measurements of small-angle scattering and backward diffraction.

At the iMATERIA instrument (Fig. 1 ▸), 1500 tubes of ^3^He detectors are arranged to view the specimen, in five detector banks that have a view over a wide scattering angle range, including small angle (∼0 to ±15°), low angle (∼20 to 40°), 90° (∼70 to 110°) and backward (∼150 to 180°). This detector arrangement provides a wide range of scattering angle 2θ and is combined with a pulsed neutron beam that has the wavelength bandwidth λ = 1–10 Å. The value of λ for a pulsed neutron beam is determined by a TOF method, and the wavenumber *q* is determined using the following equation:
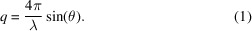
The iMATERIA instrument covers the full range 2θ = 0 to 180° (Koizumi *et al.*, 2020 ▸). Instantaneous multi-scale observation (multiple wavenumber range 0.007 < *q* < 30 Å^−1^) is possible by combining the scattering angle dispersion law and the energy dispersion law.

**Figure 2:**
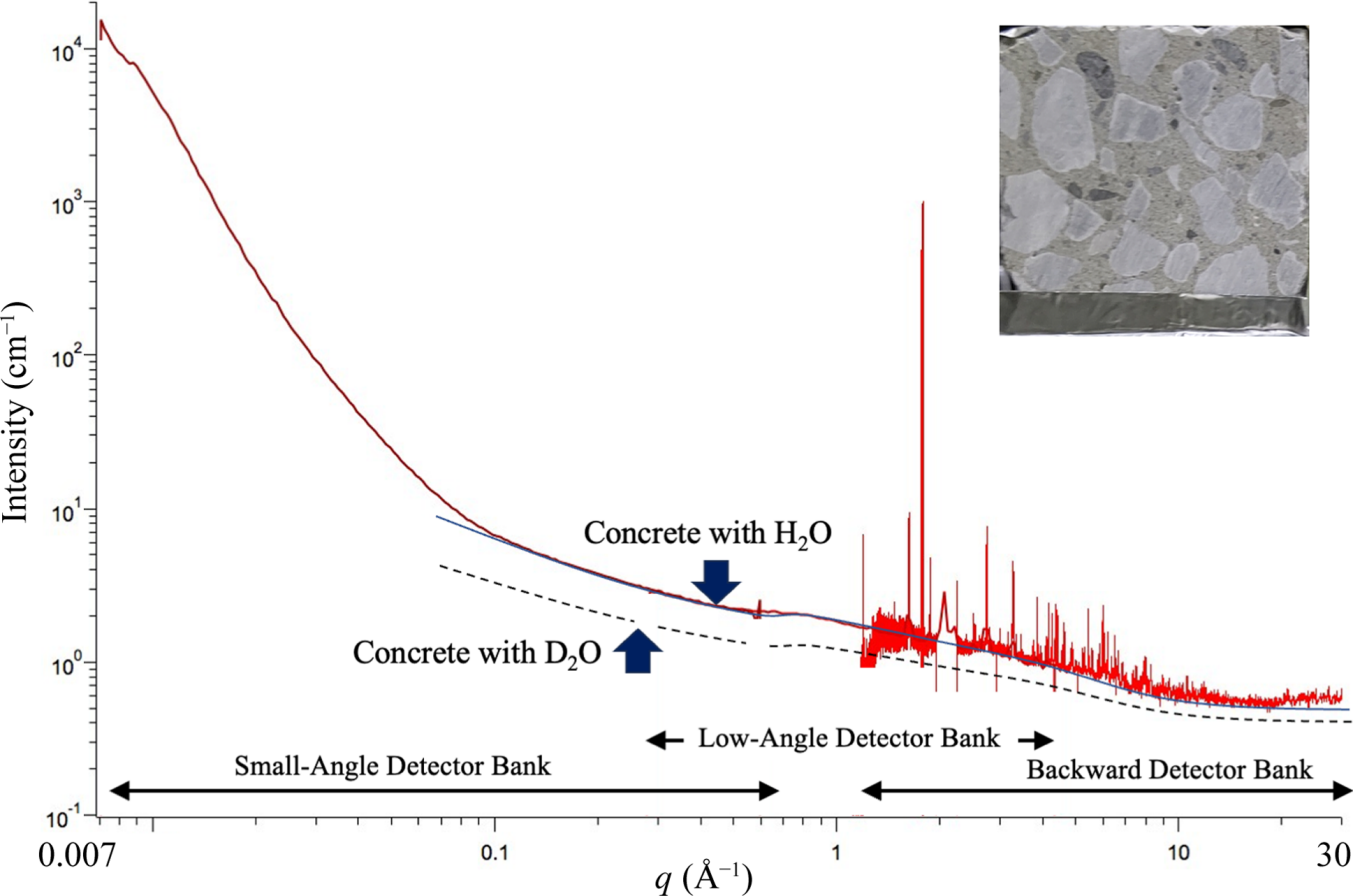
Multi-scale observation of concrete materials containing cement, sand and crushed rocks (Kubota *et al.*, 2025 ▸).

Fig. 2 ▸ shows an example measurement of concrete, provided by Wakachiku Kensestu Co. Ltd, Japan (Kubota *et al.* 2025 ▸). The incident neutron beam is transmitted through a slab-shaped concrete sample and observed by a distant 2D detector. A wide range of *q* can be detected in one shot in several minutes of measurement time, ranging from diffraction due to crystal structures in the concrete (*q*_max_ = 30 Å^−1^) to small-angle scattering such as that due to crystal grains, aggregates of sands or stones (*q*_min_ = 0.007 Å^−1^). A background of incoherent scattering from hydrogen is also recognized under the coherent scattering that contains this structural information. By replacing water with D_2_O, the incoherent scattering decreases as a factor of 1/10 (cm^−1^). The iMATERIA instrument can achieve *q*_min_ as small as 0.002 Å^−1^ by using the fifth, ultra-small-angle detector bank (Fig. 1 ▸). This value is the standard wavenumber range of nuclear reactor SANS (monochromatic angular dispersion) (Jeffries *et al.*, 2021 ▸).

If the second pinhole immediately before the sample in Fig. 1 ▸ is made of a boron carbide sintered body (B_4_C) or pure cadmium, then backward diffraction (2θ is close to 180°) occurs from the pinhole material itself. This is not desirable for diffraction data reduction since it is observed by the backward detector. A non-crystalline slit (a B_2_O_3_–SiO_2_–Li_2_O–Gd_2_O_3_ system) that does not cause diffraction was therefore developed (Takahashi *et al.* 2025 ▸), and the beam size and scattering angles needed for small-angle scattering were selected. The diffractionless and shielding performance was increased by including additives in generic borosilicate glass.

### 2.2. Multi-time-domain observation

**Figure 3:**
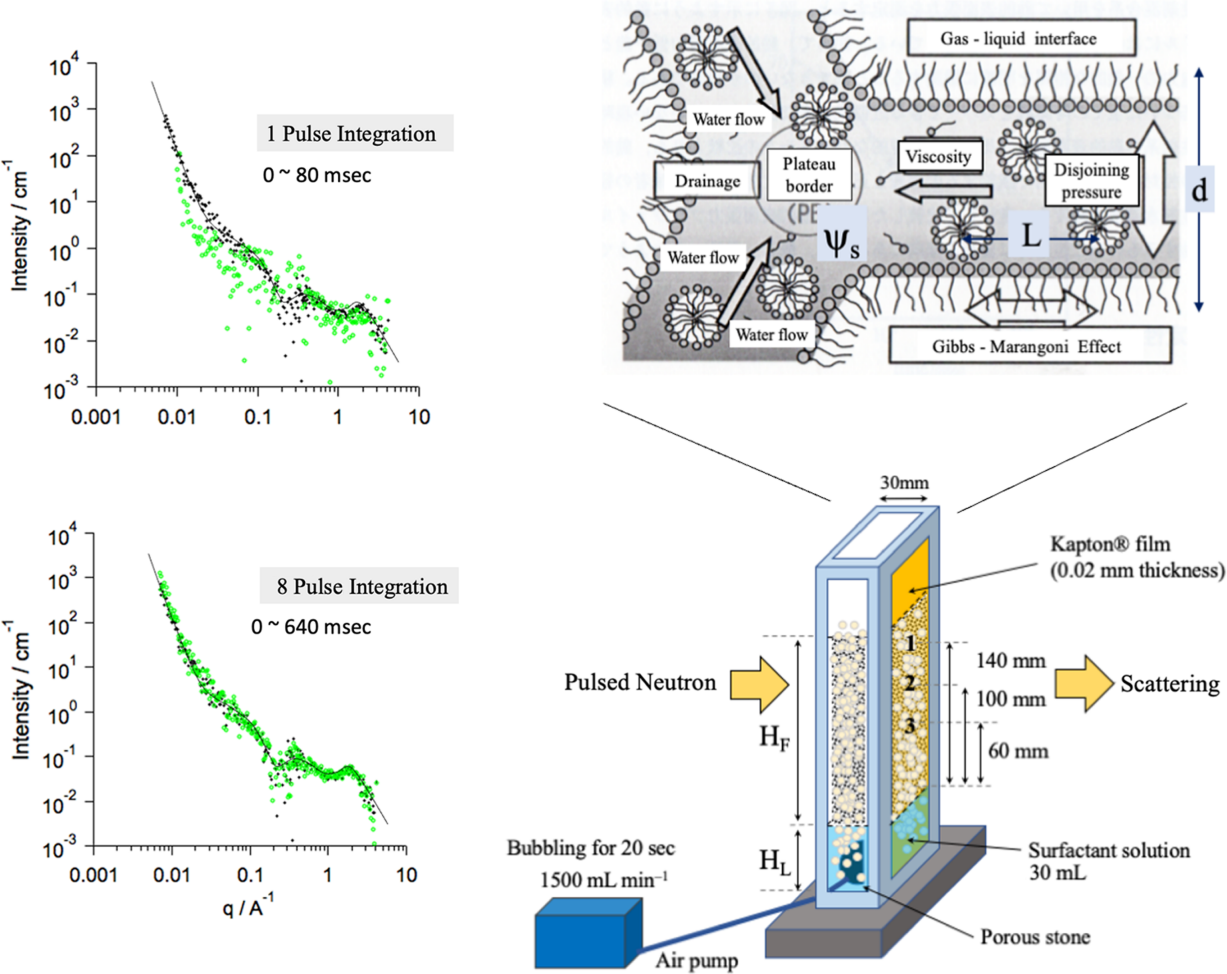
(Left) Results of time-resolved SANS measurements on foams prepared using an air-pump with a porous stone at 750 kW power. (Right) Schematics of experimental details (Koizumi *et al.*, 2023 ▸).

To demonstrate how quickly we will be able to perform a small-angle scattering measurement when the J-PARC thermal output reaches 1 MW, we use a test measurement of foam (thickness 30 mm, beam size 10 mm × 10 mm) created using shampoo surfactant [sodium do­decyl sulfate (100 mmol dm^–3^) in D_2_O] (Koizumi *et al.*, 2023 ▸). This experiment was conducted at an output of 750 kW through collaboration with Kuraray Co. Ltd. The top-left and bottom-left parts of Fig. 3 ▸ show the SANS integrated across 0.08 s, corresponding to one pulse under a double frame operation, and 0.64 s (eight pulse integration), respectively. The black dots are the results for integration for 1 s. Comparing these demonstrations shows that observation of the foam is possible at 0.64 s of data accumulation. When converted to 1 MW, this test indicates that observation will be possible in 0.5 s. Whereas the neutron moderator for the iMATERIA instrument is a poison-type, a neutron flux of 10 times this can be used at TAIKAN (BL15), which has a coupled-type moderator (Takada *et al.*, 2017 ▸; Teshigawara, 2023 ▸). In other words, time segmentation in steps of 0.05 s will be possible at J-PARC. This example shows that time-resolved observation with a wide range of time intervals (multi-time-domain) from milliseconds to, at most, several days is possible.

### 2.3. Multi-contrast observation

Commercial tires are one example of a product composed of multiple components, including carbon black, silica microparticles, ZnO co-catalyst (promoter), rubber polymer and sulfur crosslinks. Since the interaction between the rubber polymer and filler surface is directly linked to performance such as fuel consumption and braking, it is important to understand the interactions between the filler components and sulfur crosslinked polymer chains. In the structural analysis of this kind of many-component system, contrast variation, which is a specialty of neutron scattering, is particularly powerful (Koizumi *et al.*, 2021 ▸).

Contrast variation consists of methods for chemically labeling organic molecules and solvents with deuterium or physicochemical methods for polarizing the nuclear spins of hydrogen using a low temperature (close to 1 K) and high magnetic field (7 tesla), achieving 90% polarization in polystyrene (Noda & Koizumi, 2019 ▸). The latter is called ‘dynamical nuclear polarization (DNP)’, and using this method has the advantage that the coherent scattering length density (ρ) of hydrogen can be varied more than in the case of deuterium labeling. Because this does not require the chemical substitution process of deuteration, it has become established as a common daily analysis method that can observe ‘products as they are in their native state’. Since it became available in 2019 ▸ at the iMATERIA instrument, this method has been used by more than 20 Japanese companies, including Sumitomo Rubber Industries Ltd (Noda *et al.*, 2020 ▸).

The hierarchical structure of fiber or hair that contains water has also been studied by a multi-contrast experiment using the DNP method (Stuhrmann, 2007 ▸). The results of this structural analysis are useful for industrial problems such as understanding the drying behavior by hairdryers or the penetration of permanent dyes and the effect on higher-order structures.

**Figure 4:**
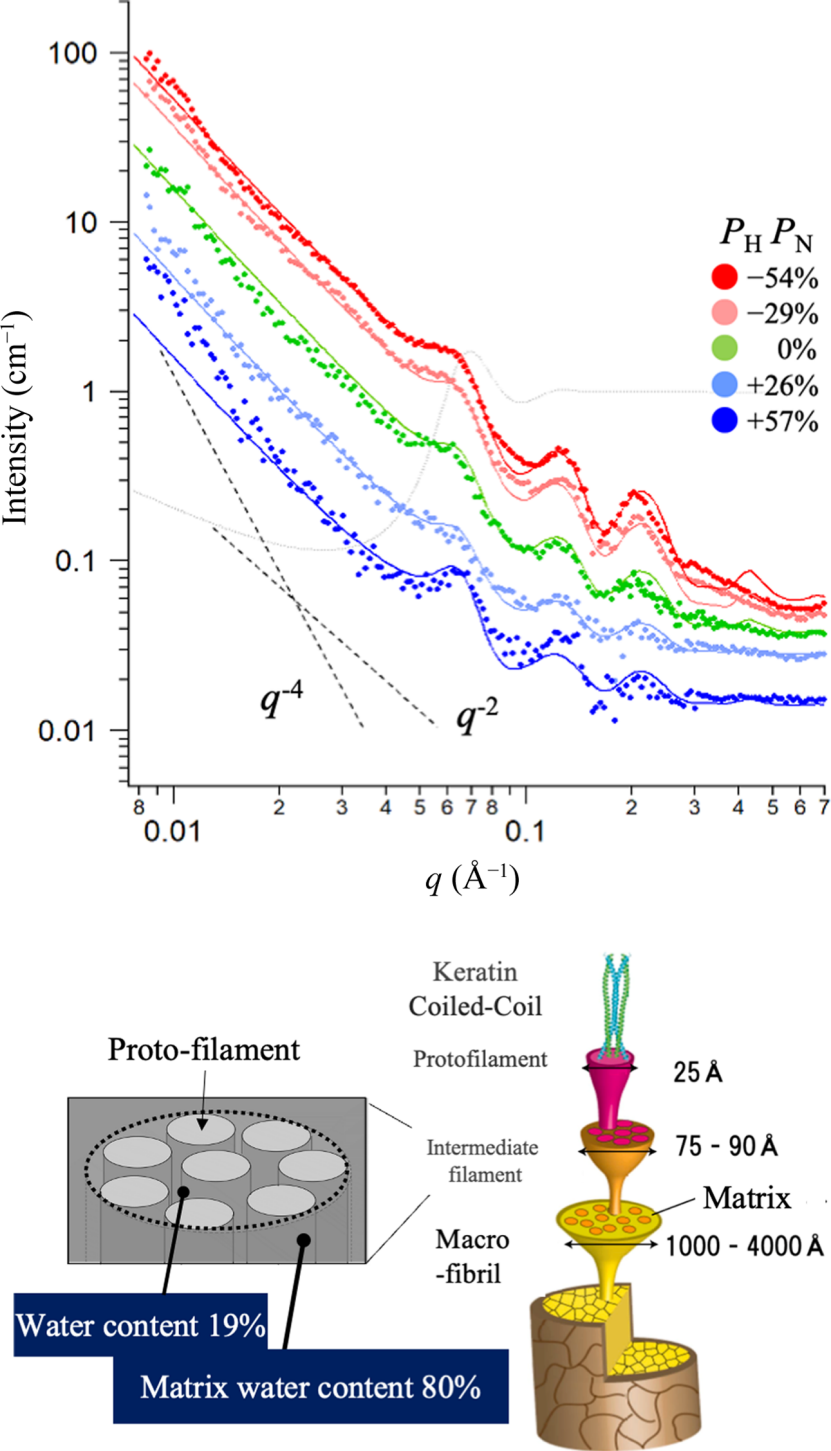
SANS profiles obtained from human hair by changing the scattering contrast Δρ through DNP (top). Schematic of the hierarchical structures in a hair (bottom). SANS observes the intermediate filament and water content (Noda *et al.*, 2023 ▸).

As shown in Fig. 4 ▸, the size measured in real space by SANS was 1 to 600 nm, and this supports the presence of structures such as protofilaments with a hierarchical structure like hair and intermediate filaments. Note that the positive polarization of P_H_P_N_ > 0 gives a decrease of incoherent scattering, where *P*_H_ and *P*_N_ refer to polarization for hydrogen and neutron, respectively. The morphology where intermediate filaments form a hexagonal lattice in a protein matrix swollen by water is described by the scattering function of a paracrystalline model (Hosemann & Bagchi, 1962 ▸). In the protein matrix containing water, Lorentzian small-angle scattering occurs, similarly to the earlier crosslinked network. Furthermore, microfibrils, which are a higher-order hierarchical structure, and the cuticle cell membrane complex cause Porod scattering (*q*^−4^). To reproduce the observed small-angle scattering of hair using these multiple contributions, the relative intensities of the three scattering functions were determined while calculating the contrast factor (difference in coherent scattering length density Δρ) by taking the water content into account. Since scattering intensity is linked to the paracrystalline structure parameters (lattice size, spacing between filaments *etc.*), the small-angle scattering needs to be obtained and discussed on the basis of multiple contrasts (Δρ) using DNP. The DNP method offers the advantage that structural analysis can be performed without confounding differences between individual samples since it allows the contrast to be varied for the same hair sample. This is an example of utilizing multi-contrast for determining the domain scattering function that reproduces a sample.

However, to determine the spatial distribution of the components that make up a sample, it is also useful to perform decomposition into the partial scattering functions of each component. One example is the structural analysis of a multi-network elastomer developed with the aim of easier recycling (Iwasaki *et al.*, 2025 ▸). DNP, which is operated by PC automatically, changes the scattering length density of hydrogen, which is covalently bound with molecules in a continuous and reversible way during SANS measurements. Thus this method enables us to analyze the domain components by finding matching points using continuous change in the scattering length density of domain components.

Let us now consider more features of SANS obtained for polymer materials in relation to using contrast variation. Non-crystalline (or partially crystalline) polymers that have a liquid-like ‘anisotropic structure have appeared in products. In such cases, the small-angle scattering can be given in terms of the Debye scattering equation (Debye, 1933 ▸) as follows:
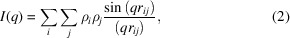
where ρ*_i_* of the *i*th molecule is the mean scattering length density and *r_ij_* is the distance between the *i*th and *j*th mol­ecules.

This is the result of performing phase calculations under a random orientation condition to determine the scattering intensity by treating the disordered structure as isotropic. Note that the Debye representation also appears in the calculation of spherically symmetric domains. Since the Debye scattering equation is a function of the scalar wavenumber *q*, a situation occurs where there is insufficient information for precise structural analysis of multiple components. In the case of reflectivity from multilayers, the **q** direction is parallel to the normal vector of the multilayers. This is why precise analysis is possible without changing the contrast for reflectivity measurements of multilayer films or for single crystals, giving the periodicity of layers of atoms or the translational symmetry of a unit cell, respectively. Recovering additional information can also be achieved by contrast variation. The Debye description of equation (2) is the same as the total scattering from amorphous structures or the powder diffraction for metals. However, in the case of powder diffraction, individual diffraction peaks at different *q*-values supply enough information to solve a structure having multiple elements. Note that combined use of electron microscopy is also useful for recovering additional structural information (Koizumi *et al.*, 2019*b* ▸; Koizumi *et al.*, 2019*c* ▸).

### 2.4. Multi-sample observation

**Figure 5:**
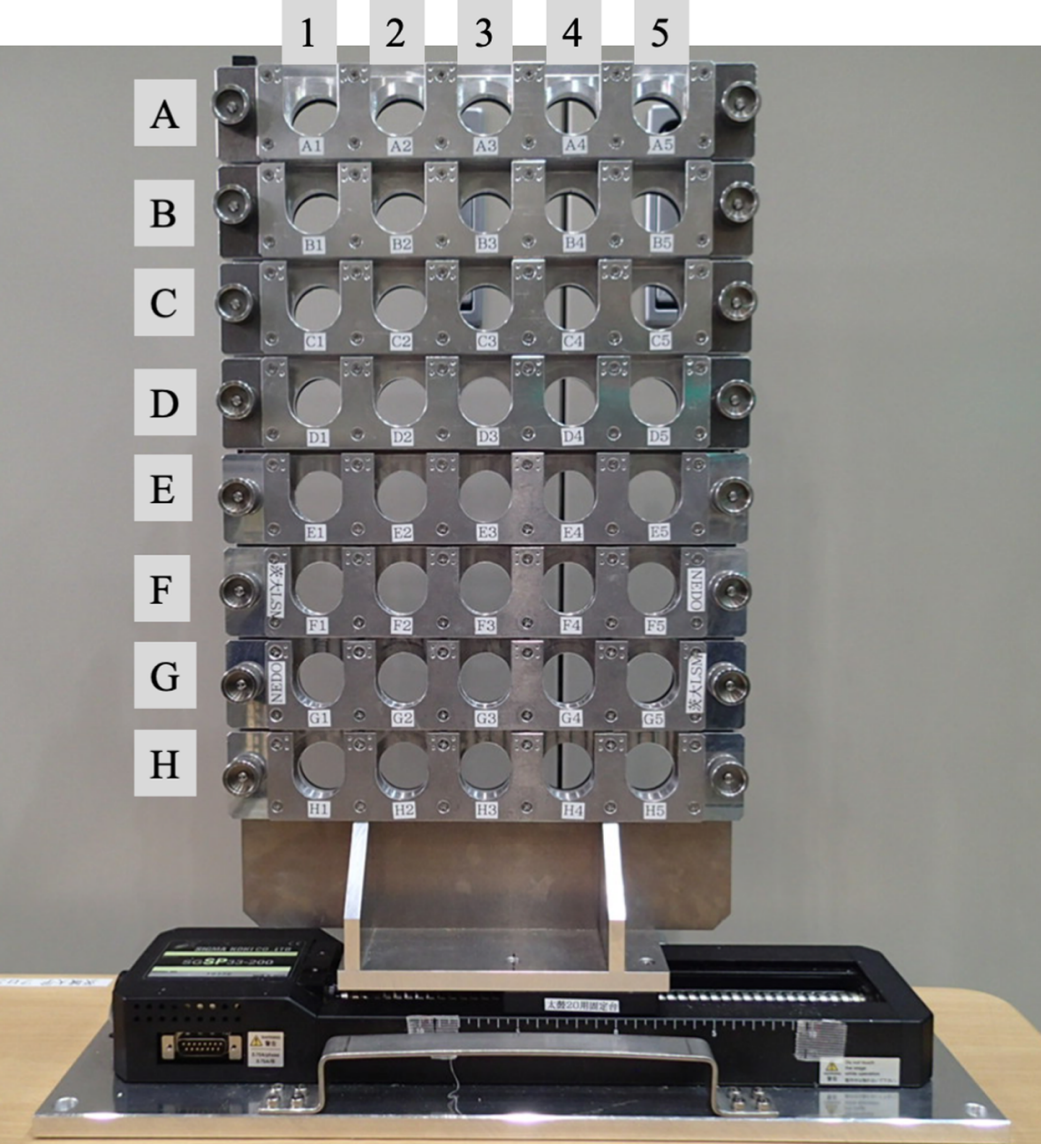
Automatic sample changer for handling 40 specimens in SANS measurements.

**Figure 6:**
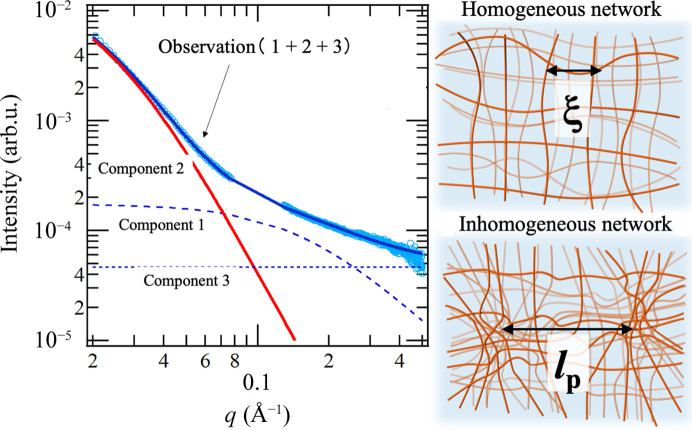
(Left) SANS profiles obtained from the crosslinked ethylene propylene diene monomer rubber, swollen by toluene. (Right) Schematics showing homogeneous and heterogeneous crosslinks of polymer chains.

Next, let us look at examples of practical use of SANS in industry. One successful example is multi-sample observation by Mitsui Chemicals Inc. (Mita, 2019 ▸). SANS was utilized in the development of a metallocene catalyst that enables uniform polymer crosslinking. A large number of polymer films (about 100) were prepared with different crosslinking conditions by the synthesis group in charge of catalyst development. These films were examined by automatic SANS measurement using the automatic sample changer (Fig. 5 ▸) of the iMATERIA instrument. The measurement time was around 24 h for 100 samples. Screening of the films was then performed to judge whether the crosslinking was homogeneous or not by focusing on the magnitude of the intensity of the small-angle scattering at the smallest *q* (Fig. 6 ▸). The crosslinked films were swollen by a good solvent (toluene) in order to visualize the heterogeneity according to the ‘frozen blob’ model (Bastide & Leibler, 1988 ▸). The screening results were fed back to the synthesis group and the products were successfully commercialized.

The screening method illustrated in Fig. 6 ▸ is formularized as follows. In the case of a uniformly crosslinked gel, the small-angle scattering is given by a Lorentzian form (component 1 in Fig. 6 ▸) and the correlation length ξ corresponds to the mesh size. When the crosslinking structure is non-uniform, as shown in Fig. 6 ▸, Debye–Bueche-type small-angle scattering (component 2 in Fig. 6 ▸) is further added on top of component 1. There is also incoherent scattering (*I*_inc_, component 3). These are formulated as follows (Onuki, 1992 ▸; Panyukov & Rabin, 1996 ▸):
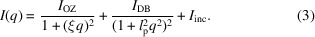

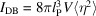
, where *l*_P_ is the correlation length, *V* is the scattering volume and 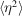 is the ensemble average of the square of the density variation. Integrating the Debye–Bueche-type small-angle scattering *I*_DB_(*q*) of component 2 across the entire wavenumber space gives
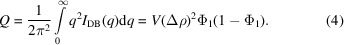
This integral result is called the invariant *Q* and is given by the volume fraction Φ_1_ of a densely crosslinked phase and the contrast Δρ compared with the uniformly crosslinked phase. If the crosslinking is uniform, then 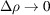 and 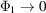, and we get only the Lorentzian-type small-angle scattering of com­ponent 1. By following this scenario, SANS, providing structure information, was linked with the field of catalyst development in the chemical company. The advantage of neutron beams compared with X-rays of 0.1 mm beam size is that the mean over an ensemble as determined with a beam size of diameter 10 mm can be observed, and the mesh structure of the polymer is not damaged by irradiation with neutron beams.

In other words, the *Q* value discussed here is the apparent scattering cross-section Σ_sca_ (= *Q*), taking into account the observation range determined by the experimental conditions. The apparent transmission (*T*_s_) is given by the following using the scattering cross-section Σ_sca_ and film thickness *d*:
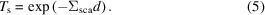
On this basis, we expect that it is possible to perform screening of the quality of crosslinked structures by focusing on the magnitude of the transmission. This task will be achieved at an instrument originally designed for neutron radiography (imaging), too.

**Figure 7:**
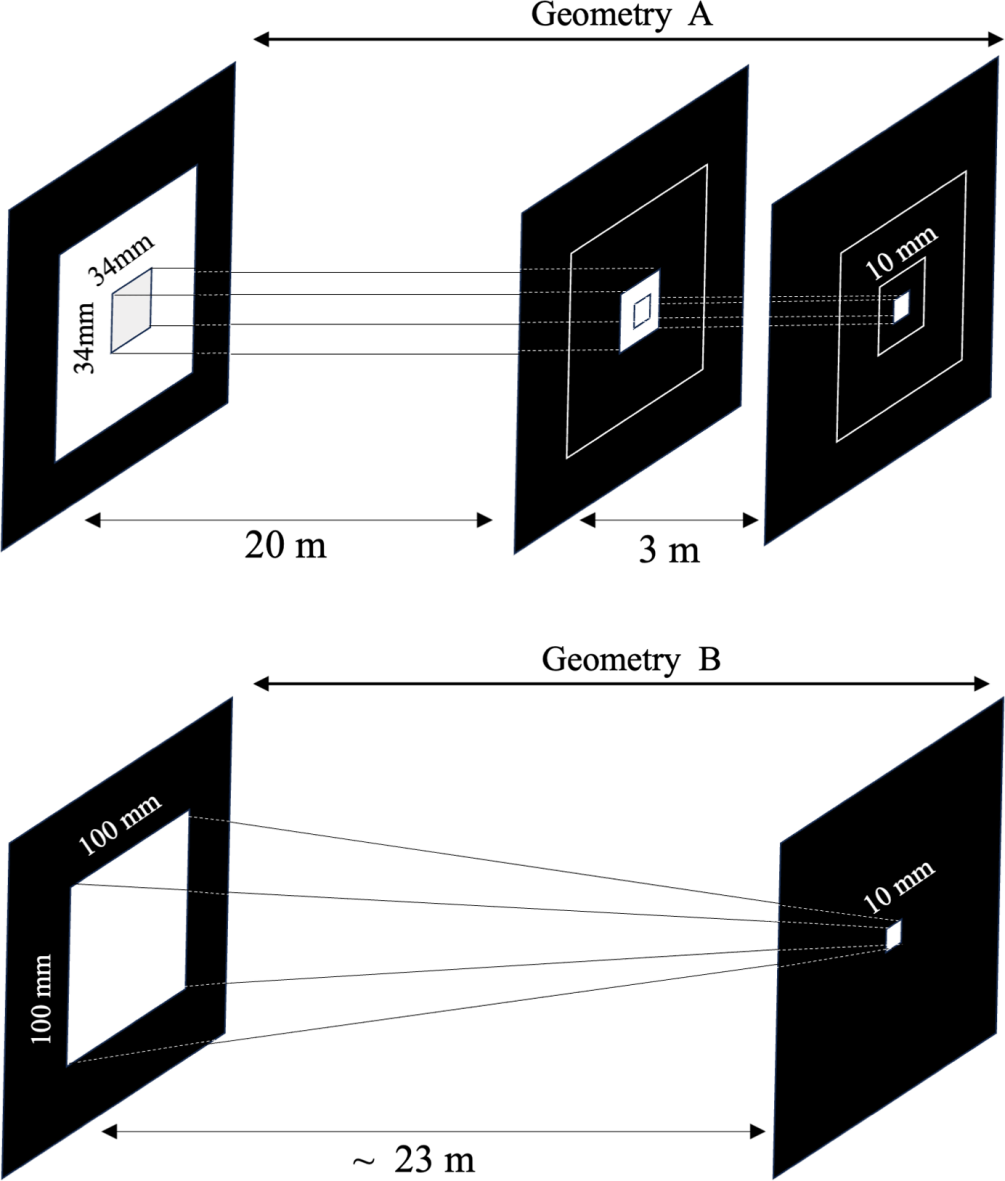
Schematics of the beam sizes along a beamline (top) with and (bottom) without a guide tube.

Let us attempt to calculate the usage efficiency of the neutrons in this experiment. At the iMATERIA instrument, the beam aperture immediately after the moderator is 100 mm × 100 mm, and after this there is a neutron guide tube with a cross-section of 34 mm × 34 mm, which leads the pulsed neutrons to the instrument (Fig. 7 ▸, top). In SANS experiments, a neutron beam with a cross-section of 10 mm × 10 mm is shaped by a pinhole before reaching the sample. Generally, the thickness is adjusted until the transmission of the film is approximately 70%. This is necessary to suppress multiple scattering and inelastic scattering from hydrogen.

Under these conditions, we compare the geometries A and B in Fig. 7 ▸, where the difference is the size of the first aperture. The usage efficiency ɛ of the neutron beam is 3.5% {= [(34 mm × 34 mm)/(100 mm × 100 mm)] × 0.3 × 100}. This means that 96.5% of the arriving neutrons were either removed by the slit without being used or transmitted through the sample and absorbed by the damper. If we take this discussion further back to include the generation source such as the upstream moderator, then we expect the utilization efficiency to be even lower.

Let us assume that the measurements conducted for Mitsui Chemicals Inc. mentioned above are also possible by taking the ‘transmissivity’ as an indicator of crosslinking. We therefore inserted a vacuum tube as shown at the bottom of Fig. 7 ▸ such that the incident neutrons were received through two holes of size 100 mm × 100 mm at the incident aperture and 10 mm × 10 mm at the sample position. Here, should we not expect a benefit of more than 100-fold over the current state from a comparison of the pinhole area ratios? We calculate that the 24 h of multi-sample observation conducted by Mita (2019 ▸) as described above can be completed within 15 min (= 24 h × 60 min/100 times).

On the basis of the same idea of using a large incident beam, the ib-SAS small-angle scattering instrument was constructed at the RANS compact neutron source at RIKEN to keep the neutron utilization at the maximum limit by directly mounting multiple pinholes to cover the entire surface of the approximately 150 mm^2^ square moderator (Koizumi *et al.*, 2025*b* ▸). In a future large facility, beam shaping for such a large area will be determined according to user choice.

At the iMATERIA instrument, a second sample port has been made immediately after the small-angle scattering detector and immediately before the beam damper with the aim of efficient neutron beam utilization, and the beam is being used for various purposes. Our future plan is to develop an ultra-small-angle scattering detector to reach to the *q* = 10^−4^ Å^−1^ wavenumber range. We are also planning to perform prompt γ-ray measurements as described below as a way of using neutrons unrelated to small-angle scattering [see Fig. 16(*b*)].

### 2.5. Multi-angle observation

**Figure 8:**
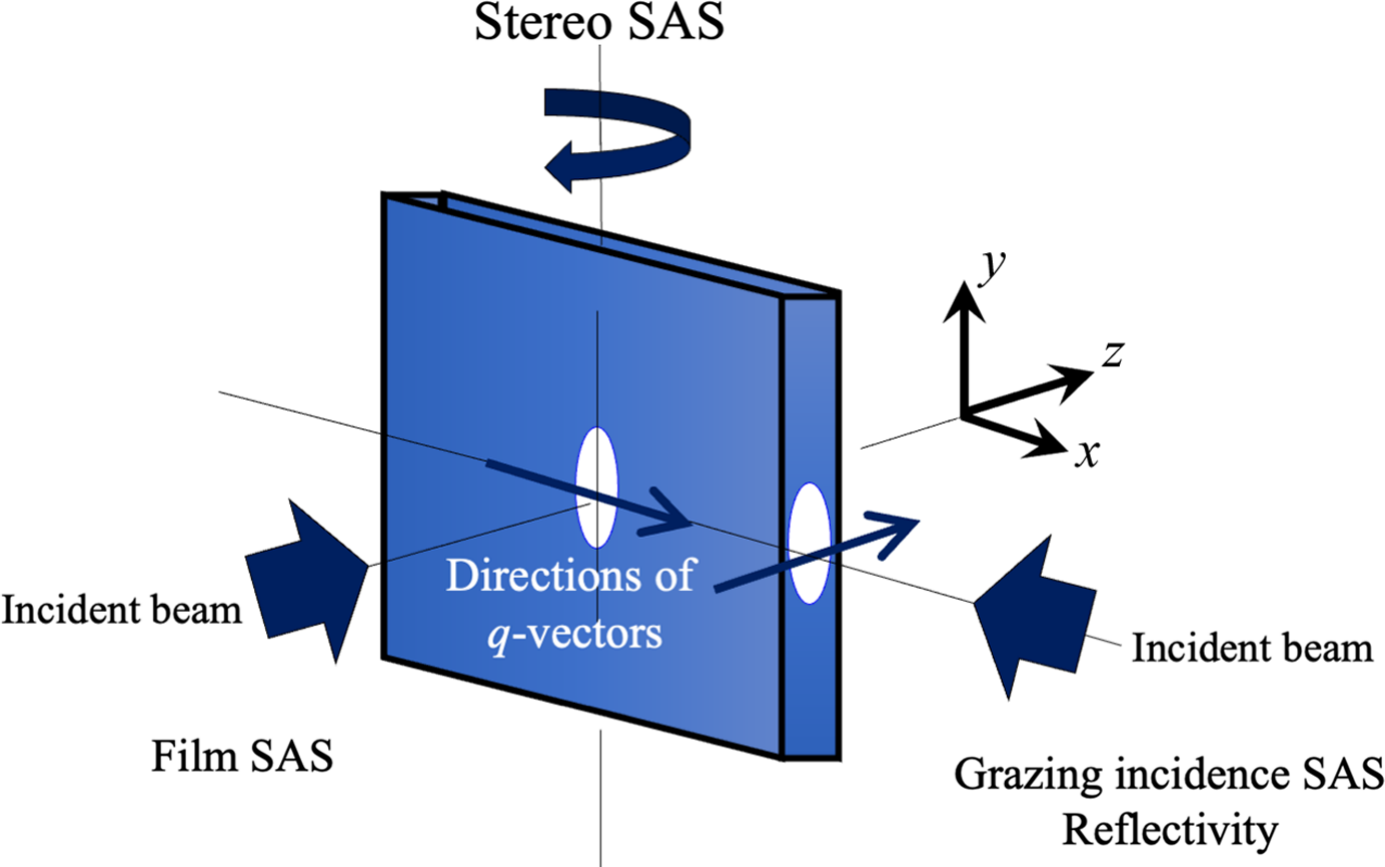
Multi-angle observation of a film specimen, including conventional SANS and surface reflectivity.

The industrial products supporting our daily life often have a film or plate shape. The interior (bulk) of a film has various mechanisms contributing to its mechanical properties, and the surface is the frontline that forms a subtle functional field that gives the handling texture, antibacterial properties, adhesion and adsorption. Usually in SANS, the neutron beam is incident from the film normal vector direction and the transmitted signal is observed (as shown for small-angle scattering from a film in Fig. 8 ▸). In contrast, in the grazing-incidence method, the neutron beam is incident onto the film surface from a grazing angle, and reflections and grazing-incidence scattering from the surface are observed. Up to now, small-angle scattering and reflectivity measurements have been performed independently on separate beamlines. For industrial users, performing these techniques on one beamline would be more economical.

**Figure 9:**
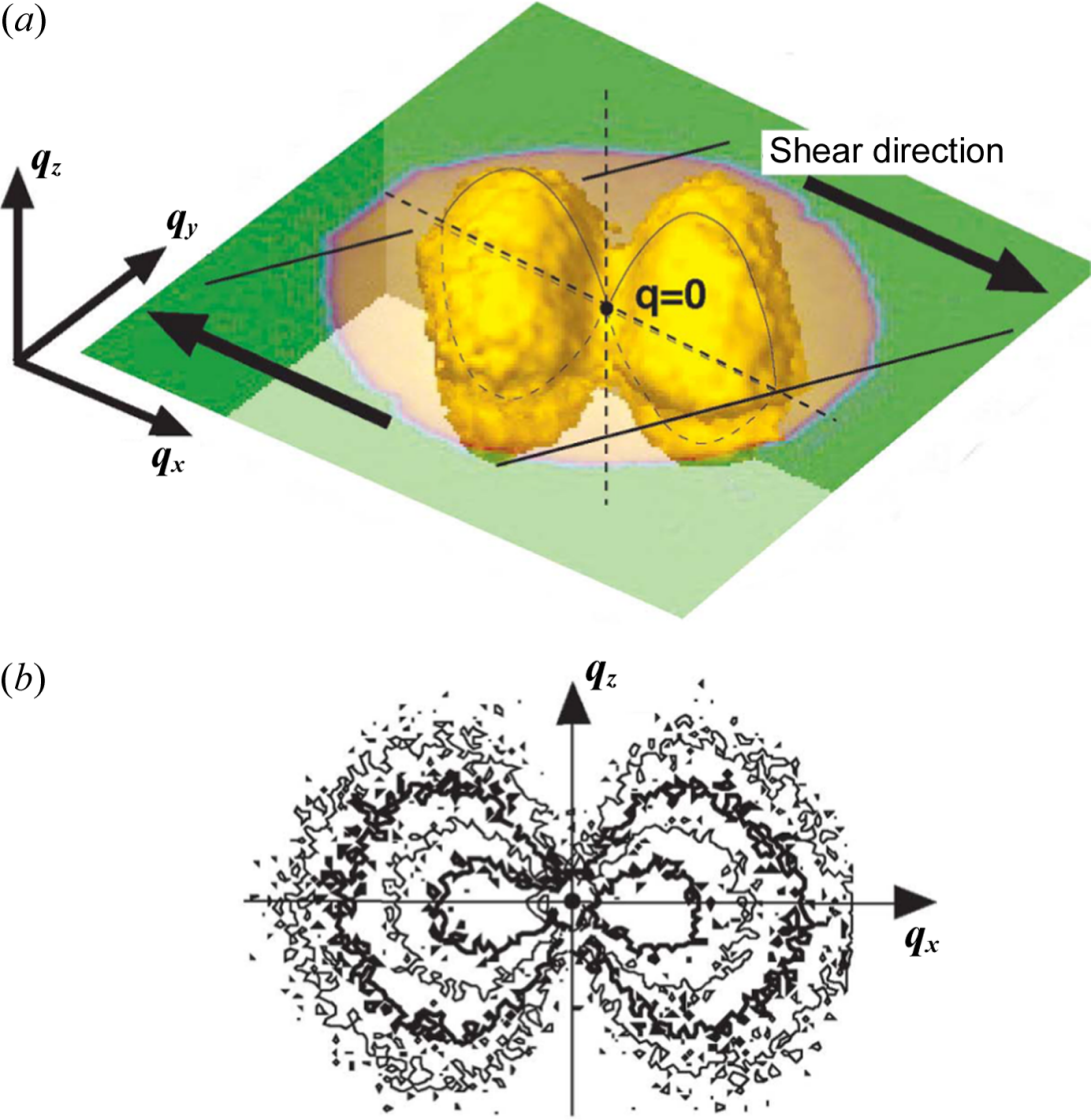
Shear-induced phase separation observed by multi-angle SANS observation. (*a*) 3D iso-intensity surface. (*b*) Section-cut view at *q_x_* = 0.

At the SANS instrument SANS-J-II at the research reactor JRR3 (Agamalian & Koizumi, 2011 ▸; Koizumi *et al.*, 2007 ▸), by rotating the film with respect to the incident neutron beam, the 3D internal anisotropic phase separated structure was determined in a dynamically asymmetric polymer blend film [polystyrene/poly(vinyl­methyl­ether)] to which shear deformation had been applied (Fig. 9 ▸) ▸ (Koizumi & Suzuki, 2006 ▸). This is called 3D SANS. The experimental results exhibited an abnormal butterfly pattern and were a good match with the theoretical ‘coupling model between local stress and density fluctuations’ (Doi & Onuki, 1992 ▸). As shown by this example, film products created through compressive and tensile machining processes have an isotropic microstructure and it is undoubtedly important to understand this in three dimensions.

As the film angle is made even shallower in 3D SANS, reflections from the film surface eventually begin to be observed. Both surfaces of the film bulk, facing to air, have a Fresnel interface, and the reflections from these surfaces strongly depend on the atomic composition of the surface and the roughness morphology. These factors are indicators that characterize the functional field of the product surface. In particular, the critical wavenumber *q*_c_ of total reflection is given by
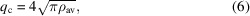
where ρ_av_ is the mean scattering length density at the surface and depends on the composition of the film surface according to the following equation:
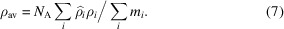
Here, *N*_A_ is Avogadro’s number, and 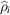, ρ_*i*_ and *m_i_* are the weight density, scattering length density and molecular weight, respectively, for the *i*th molecule.

**Figure 10:**
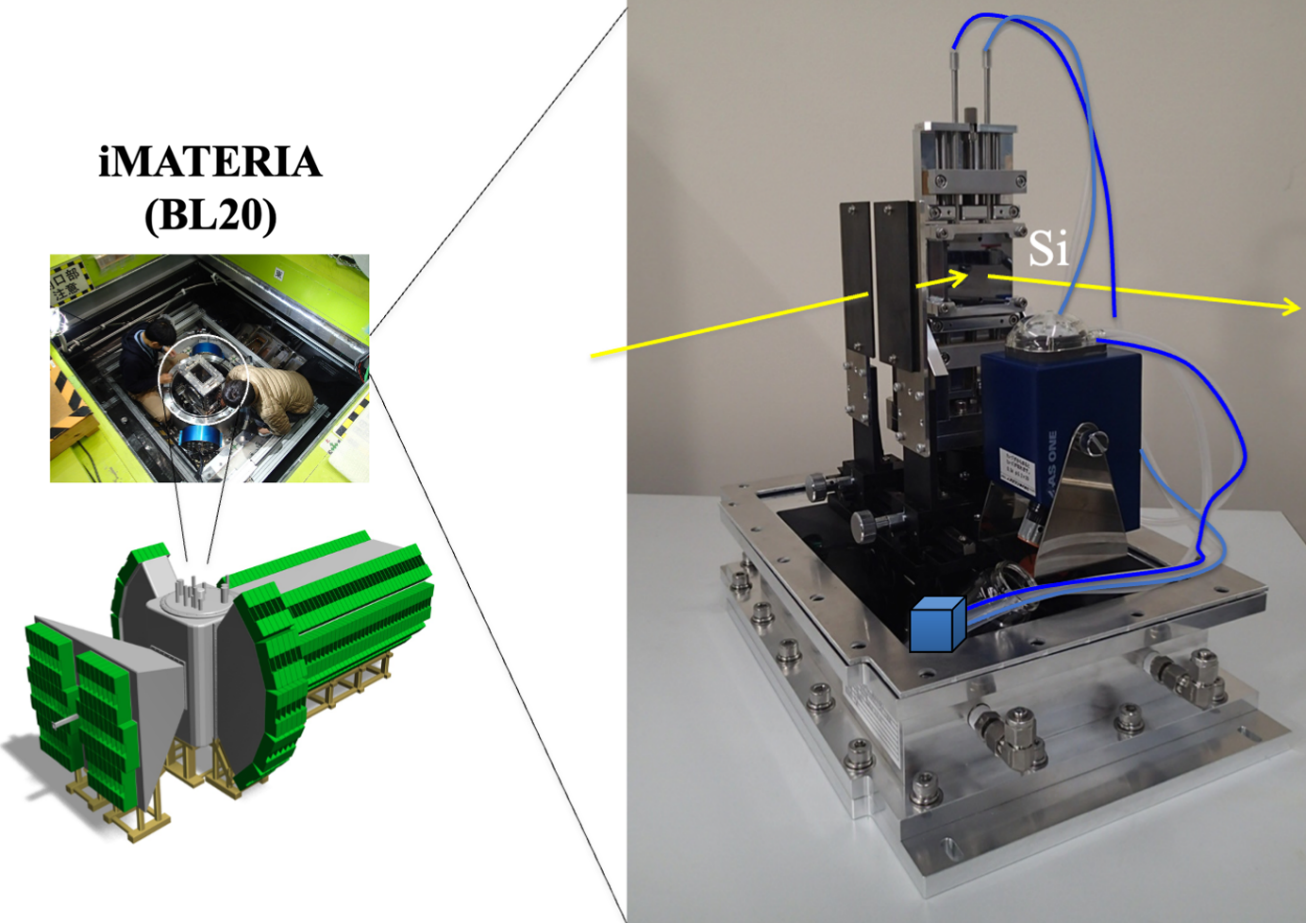
Reflectivity measurement setup (vertical stage for water exchange) performed on the iMATERIA instrument.

**Figure 11:**
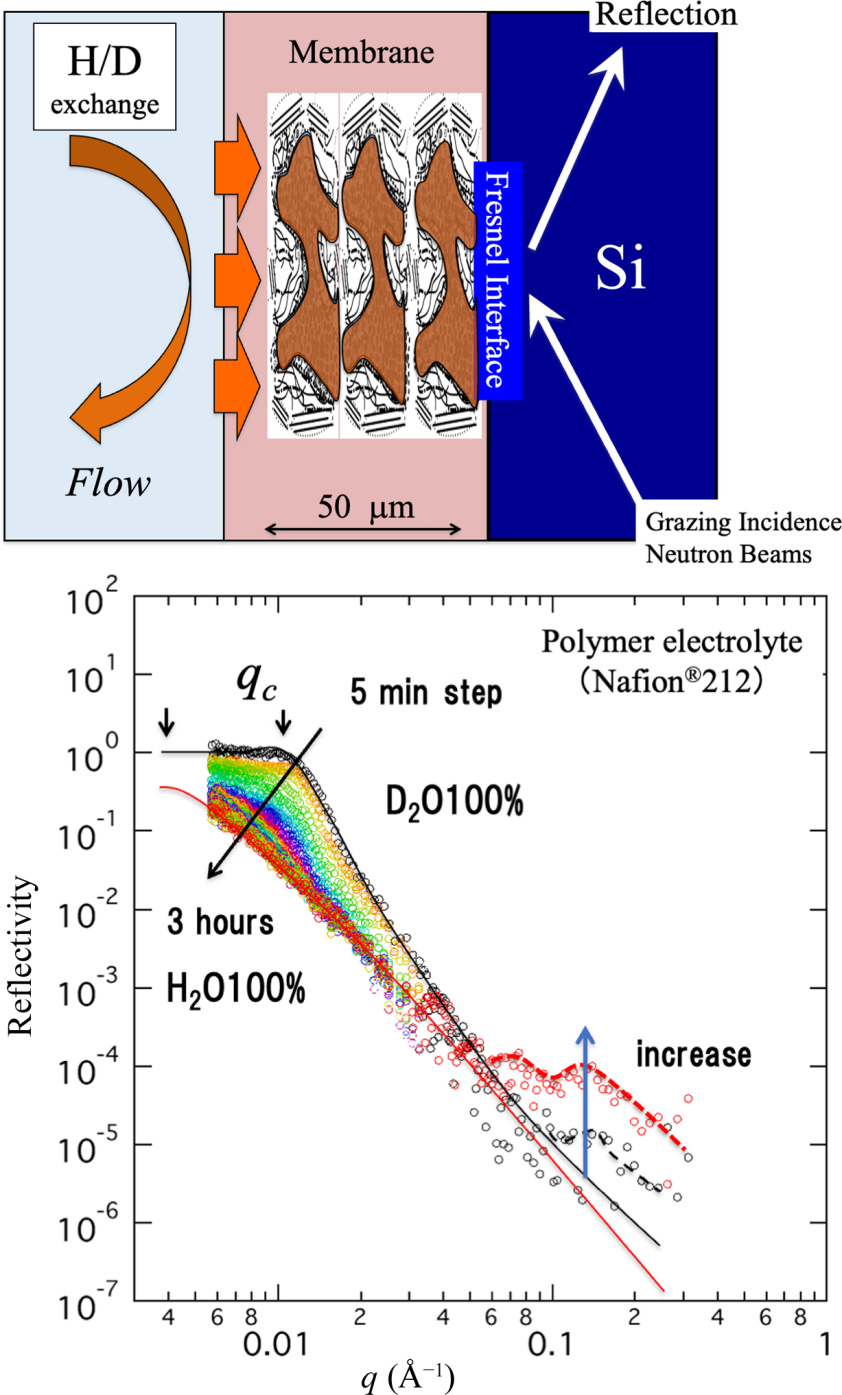
Water-exchange experiment for the polymer electrolyte Nafion. (Top) Schematic showing the total reflection observed on the Si-attached surface. (Bottom) Time-resolved total reflection during D_2_O exchange (Koizumi *et al.*, 2018 ▸).

First, let us look at an example involving the investigation of a polymer electrolyte film, which is an essential fuel cell material. For Nafion film, water diffusion across the film thickness direction was tracked by total reflection composition analysis (Koizumi *et al.*, 2018 ▸). Fig. 10 ▸ shows a portable reflection setup. For a film that has been saturated with light water (H_2_O) in advance, replacement of the water occurs when one side is immersed in heavy water (D_2_O) and it eventually reaches the opposite side. The opposite surface was affixed to a silicon substrate (Fig. 11 ▸, top). The idea is that, when a neutron beam is incident from the silicon side and the time at which there is a change in the total reflection behavior at the Fresnel interface is observed, the diffusion coefficient of water can be evaluated. As a result, it was found that there are two types of water diffusion coefficients with different magnitudes, corresponding to unconstrained fast water and slow water tightly bound with SO_4_^2+^ groups (Koizumi *et al.*, 2018 ▸) .

**Figure 12:**
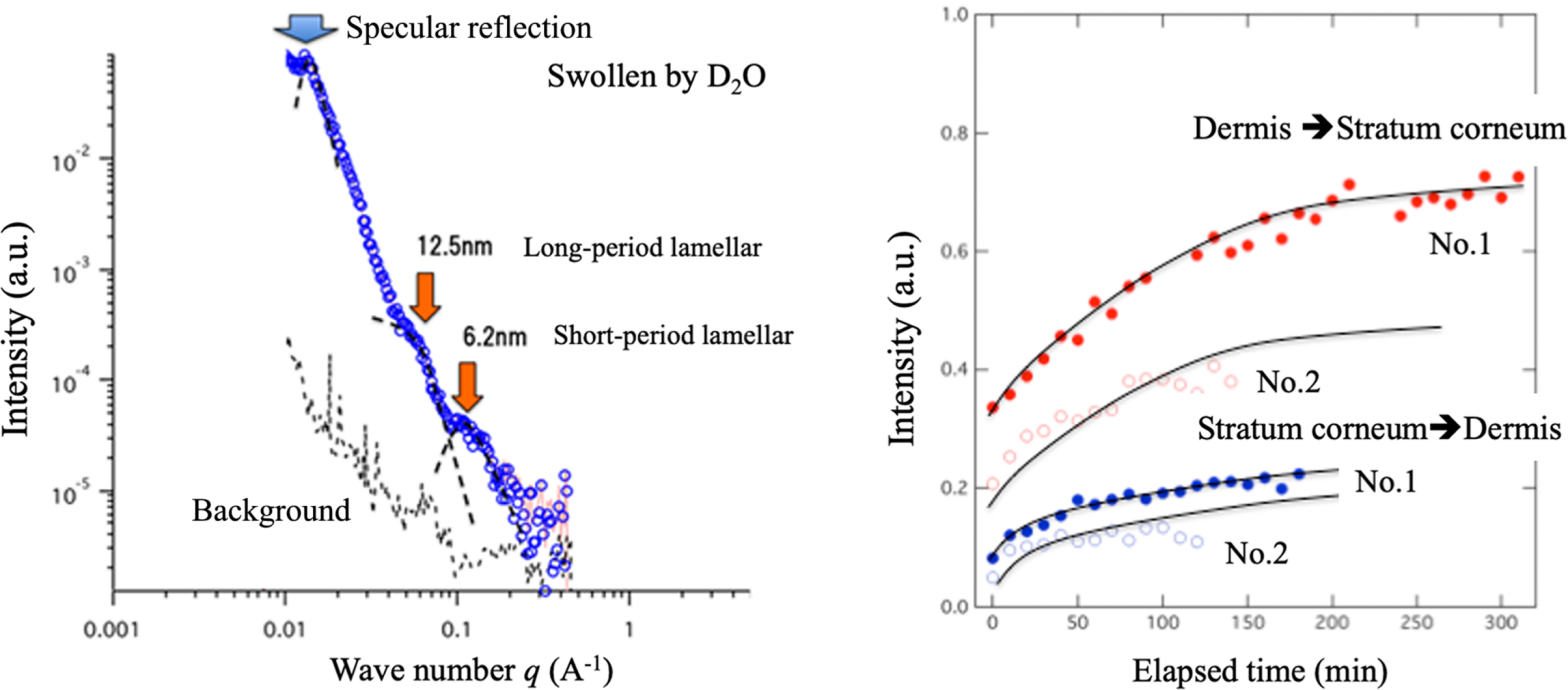
Total reflection observing skin adhered on an aluminium surface.

As a second example, results on human skin found that water diffusion from the stratum corneum to the dermis was slower than that in the reverse direction from the dermis to the stratum corneum (Oka *et al.*, 2017 ▸). This is an important result that indicates that the diffusion coefficient in human skin is anisotropic. Fig. 12 ▸ shows the total reflection obtained for the skin, attached on an Si plate. Neutrons were irradiated from the Si side in the same way as indicated in Fig. 11 ▸. The skin was swollen by D_2_O. The total reflection appears at *q* = 0.01 A^−1^, with an incident angle of 0.5°. As the wavenumber increases, we start to observe the microstructures near the Fresnel surface (long- or short-period lamella having 12.5 or 6.2 nm inter-distances, respectively). Fig. 12 ▸(*b*) shows the time-dependent observation, illustrating the intensity change at *q* = 0.01 A^−1^ as a function of time. In the case from the dermis to the stratum corneum, the intensity increases significantly compared with the case from the stratum corneum to the dermis. Thus an anisotropic diffusion coefficient across skin is demonstrated.

**Figure 13:**
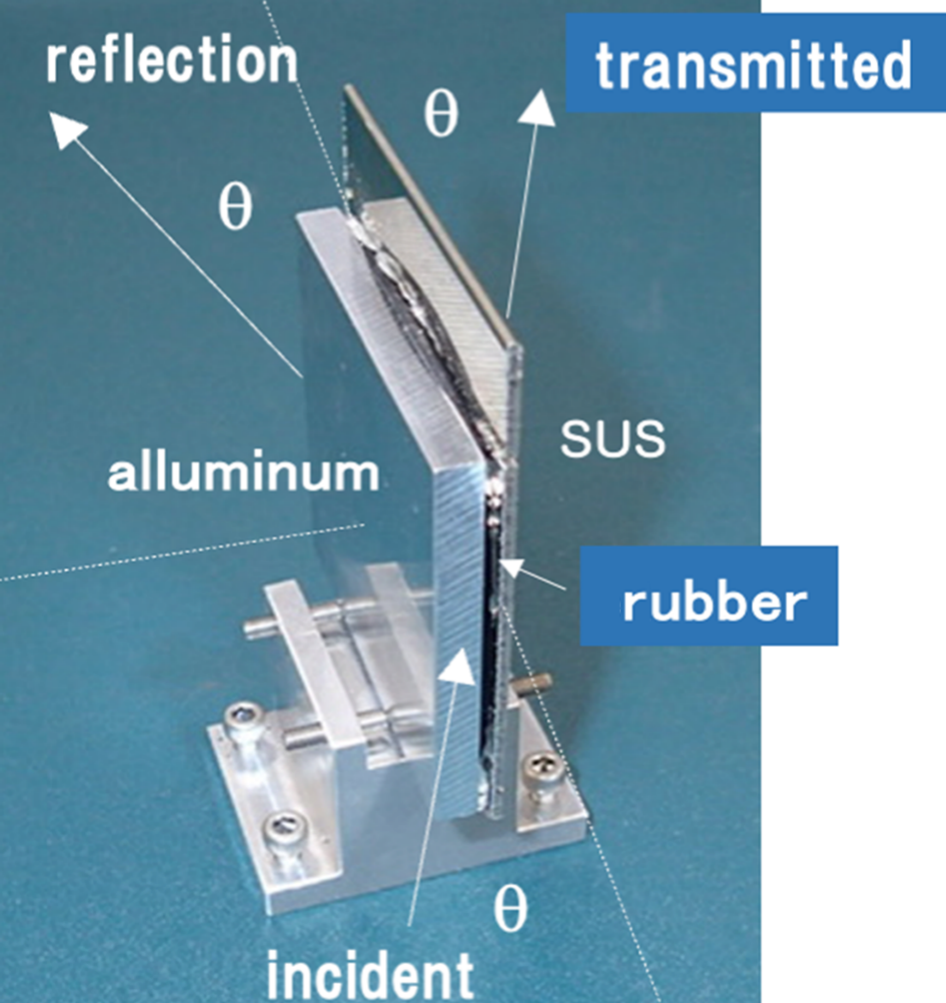
Total reflection observing rubber adhered on an aluminium surface.

A third example is related to synthetic rubber for seal packings (Koizumi *et al.*, 2025*a* ▸). Rubber has the problem that, when it is used for a long time in a high-temperature environment, burn-in onto metal surfaces occurs. The time change in the burn-in of fluoro-rubber onto a metal surface (aluminium) was observed by time-resolved total reflection composition analysis (Fig. 13 ▸). As a result, carbonization of the rubber was found to occur at the contact interface with metal due to desorption of hydrogen fluoride. Carbon nanotubes were added and evaluated as a remedy.

As a final example, the appeal of reflectometry is described from the standpoint of small-angle scattering. For the Debye representation described above in equation (2), it is difficult to precisely evaluate local structures that form at solid surfaces, such as microparticles by small-angle scattering. However, if these local structures are replaced by a stacked layer structure on a substrate, it immediately becomes possible to perform precise structure analysis (Ueda *et al.*, 2025 ▸). The idea is to make up for the limits of small-angle scattering by utilizing reflectometry.

## 3. New measurements by multi-analysis using a single beam

The products brought in from industry are composite materials made up of various parts, and the structural information obtained from highly specialized, separate, individual beamlines is limited to a single aspect of multiple products. In order to resolve these technological problems, it would be ideal to be able to perform compound neutron spectroscopy simultaneously using a single beamline. iMATERIA originally started as a powder diffraction instrument in 2008 ▸ and has been capable of performing SANS measurements since 2018 ▸. As a result, it allows multi-scale observation of spatial length scales. The goal is to combine even more different measurement methods.

Simply put, the small-angle scattering we have looked at up to this point is a transmissive optical measurement technology and resembles perspective imaging methods [neutron radiography (NR)] in this regard. Up to 2022 ▸, a simultaneous measurement system for SANS and NR was developed at the iMATERIA instrument with the support of the national research agency NEDO (New Energy and Industrial Technology Development Organization, Japan) and an Ibaraki prefecture project. The goal was a simultaneous measurement system for SANS and NR, which was initially attempted by us at the SANS-J-II instrument at JRR3, Japan, over 15 years ago (Iwase & Koizumi, 2009 ▸). Simultaneous measurement was performed by inserting and removing an NR camera immediately after a single cell of a fuel cell under operation.

Since the sample position in the iMATERIA instrument is surrounded by a vacuum, an air vessel was fabricated to enable observation of samples in air. As part of research commissioned by NEDO, a prototype single-cell movement mechanism (rotation and X-stage) was mounted on the sample stage so that a prototype single fuel cell (A4 size) of, for example, the Toyota MIRAI car product could be loaded, and an operation program was created that enabled remote operation of the stage vertical movement and movement mechanism.

An NR camera was fabricated (consisting of a scintillator, optical mirror, image intensifier and CMOS camera). The scintillator and optical mirror were optimized to be as thin as possible in order to enable simultaneous measurement with small-angle scattering. The maximum image size that can be acquired is 50 mm^2^, which is limited by the neutron guide for the iMATERIA beamline. All parts of the A4 size fuel cell can be observed by changing the height of the stage and camera by remote operation or by sliding the cell horizontally. Since the neutron image capture can acquire images every 10 ms using the TOF method, the energy (wavelength) dependence of the image can be observed. To ensure sufficient image resolution, the thickness of the scintillator was kept thin, achieving a spatial resolution of 100 µm. However, note that the spatial resolution of the image intensifier for capturing the light intensity is 30 µm.

**Figure 14:**
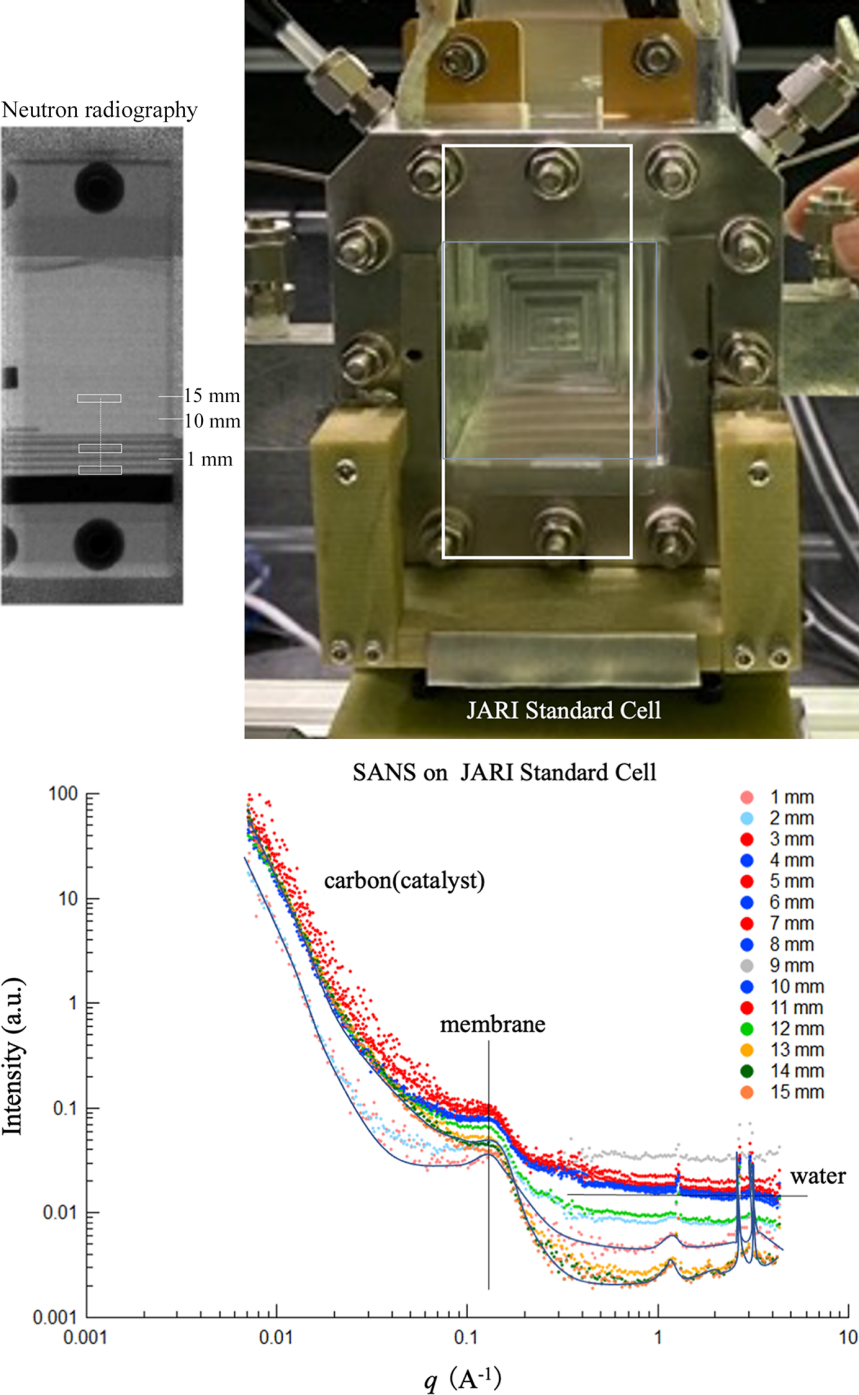
(Top) Simultaneous measurement of SANS and NR to observe the JARI reference fuel cell. (Bottom) NR image showing bulk water in a gas-flow field and SANS observed at different positions on the gas-flow field.

Observation of the liquid water in a standard polymer electrolyte fuel cell (JARI cell) was performed using the above facilities. In the JARI cell, the clumping plate was replaced with one made of aluminium, the flow channels (current collector plate) were made from aluminium and the surface was gold plated. Simultaneous measurement of NR and SANS was performed under conditions where liquid water was present in the flow channels. The top left of Fig. 14 ▸ shows a combined NR image. The liquid water motion was successfully detected as the cell temperature was increased from 80 to 105°C. The dark areas depict water that has pooled in the flow channels (flooding). The measurement range is the flow channel area of the JARI cell (white box in Fig. 14 ▸). The output of J-PARC is 850 kW and video of the liquid water moving inside the flow channels was observed in real time.

Furthermore, as shown in Fig. 14 ▸, the locations of the flow channels in the fuel cell were identified using the NR observations as a guide, and SANS was performed at each location. The location dependence was observed in steps of 1 mm from the flooded bottom area to the top area that was free of liquid water. The neutron beam size here had a height of 1 mm and width of 10 mm. The location dependence of the coherent scattering and incoherent scattering of liquid water in the catalyst layer, electrolyte layer, water channels and other locations was observed throughout the cell. Mapping information was created that overlays this structural information on the transmission video obtained by NR.

**Figure 15:**
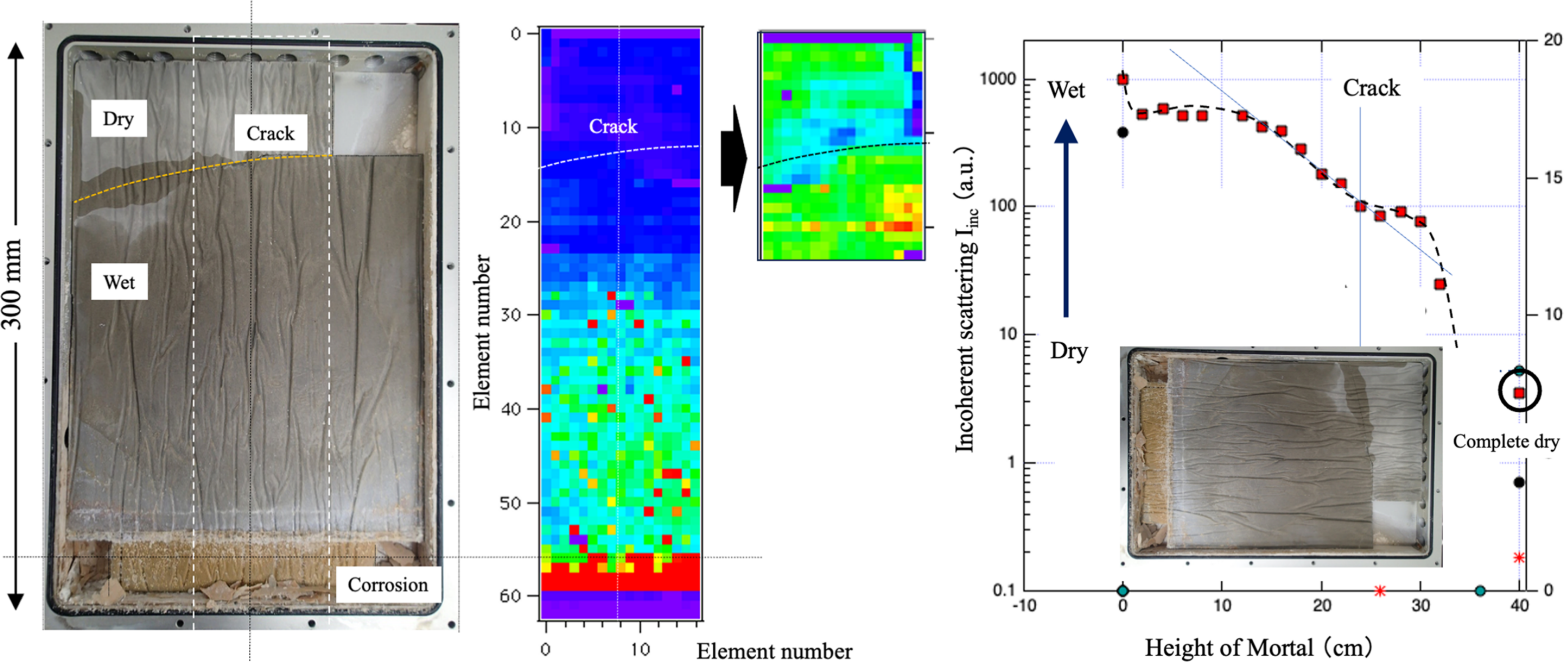
(Left) Mapping image of incoherent scattering originating from water in the mortar. (Right) Water distribution in the height direction.

Mapping imaging was proposed as part of research and development into the industrial use of compact neutron sources (RANS, Japan) and is similar to measurement by analytical scanning electron microscopy and scanning transmission electron microscopy. The difference is that neutron beams have a beam size on the order of millimetres (Fig. 15 ▸). We now introduce examples of efforts to obtain mapping imaging in relation to water absorption and corrosion of mortar. Water was added only to the bottom of a mortar sample, which was the same size as the underlying mat inside the aluminium vessel, and the water was left for a long period of time. When this was done, corrosion occurred in the bottom part, as shown in the photograph in Fig. 15 ▸ (left). The sample was irradiated with a neutron beam of size 5 mm × 5 mm, and SANS and diffraction were observed sequentially from the bottom part of the mortar. The intensity of the measured incoherent scattering was plotted as shown in Fig. 15 ▸ (middle). It was found that, as the height of the measurement increased, the intensity of incoherent scattering gradually decreased (*i.e.* the amount of water decreased) (Fig. 15 ▸, right). Similarly, we expect that the state of corrosion can be mapped by focusing on the change in the diffraction pattern accompanying corrosion. This is positioned as a non-destructive inspection technology that utilizes high intensities, and there are plans to promote this as a new method of using scattering and diffractometry.

## 4. Proposal for new neutron detection for simultaneous multi-analyses

Fuel cells operate on the basis of non-equilibrium open system conditions and are like a pulsating living organism (Koizumi *et al.*, 2019*a* ▸). For example, if the power generation state varies with time in response to flooding, then the peak scattering intensity of SANS originating from the ion conduction channels in the polymer electrolyte membrane will vary with time. This has been named the respiration mode (Koizumi *et al.*, 2019*a* ▸). When tracking such non-equilibrium phenomena, it is desirable to acquire all of the data at the site of operation. The Ibaraki Prefecture Outsource Research Cutting Edge Research (Dairy Life Field) has a plan for performing simultaneous prompt γ-ray measurement at the iMATERIA instrument as a further development towards simultaneous measurement of SANS and NR as described above.

When prompt γ-rays are considered, which correspond to the absorption cross-section Σ_abs_ of the neutron beam, the transmissivity described above is given by *T*_s_ = 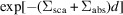. There is a plan to attempt observation of all the phenomena related to neutron beam attenuation at the same beamline. Prompt γ-ray measurements are performed at each location using a beam of millimetre size, and the distribution of a target element is mapped on the transmission image. In a non-equilibrium open system like a fuel cell, there is an outflow of material. Information about the elements in the measurement area can be attached to the structure analysis for this case.

**Figure 16:**
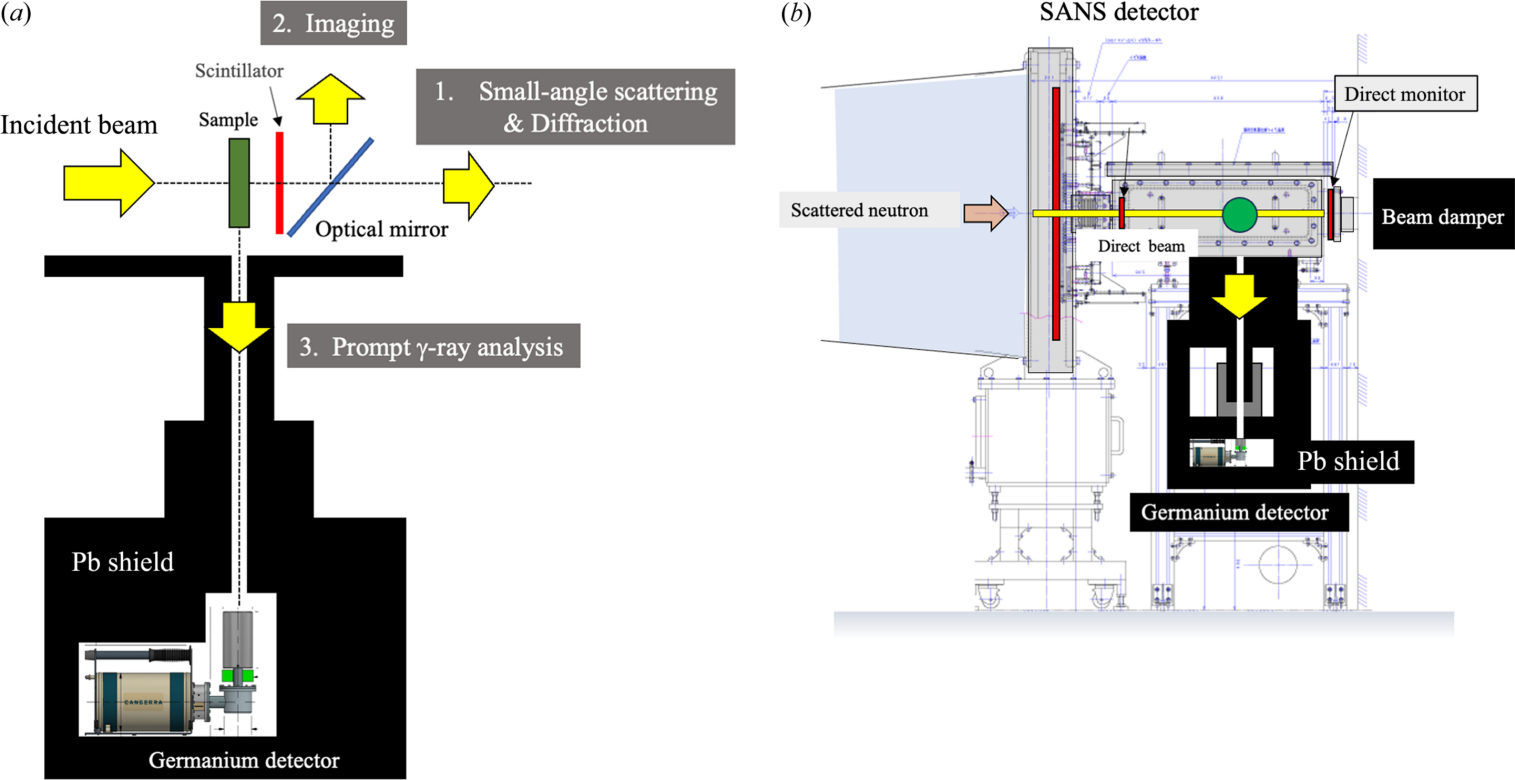
(*a*) Schematic of triple simultaneous analysis, combining SANS, NR and prompt γ-ray measurements, which will be installed at the iMATERIA instrument by the end of 2025. (*b*) Schematic of the second sample stage for prompt γ-ray measurements behind the SANS detector (future plan).

Fig. 16 ▸ shows a schematic of a triple simultaneous measurement instrument for SANS/diffraction, NR (transmission image) and prompt γ-ray measurement. The transmission image is converted to visible light by a scintillator as has been done in the past, and then the measurement is performed using a CMOS camera above an optical mirror. The prompt γ-rays emitted from the area where the beam strikes go through a fine hole in a lead shield and are detected by a germanium detector below. Examples of the detected elements include sulfur, chlorine and deuterium in industrial products. There are plans to use this measurement method for industrial problems such as the recycling of plastic materials, which was requested by the Ibaraki Prefecture Outsource Research Cutting Edge Research (Life Field). The usage is planned to start in the second half of 2025.

## 5. Conclusions

This paper describes a number of environmental use cases, based on a small-angle scattering instrument, with specific industrial requirements. There are a range of new developments described, using pulsed neutrons and TOF data acquisition, including neutron diffraction, reflectivity, imaging (radiography), prompt γ-ray analysis, and mapping images of hierarchical structural and element information.
